# Activity of *Aristolochia bracteolata* against *Moraxella catarrhalis*

**DOI:** 10.1155/2014/481686

**Published:** 2014-09-28

**Authors:** Malik Suliman Mohamed, Mona Timan Idriss, Amgad I. M. Khedr, Haidar Abd AlGadir, Satoshi Takeshita, Mohammad Monir Shah, Yoshio Ichinose, Toshihide Maki

**Affiliations:** ^1^Department of Pharmaceutics, Faculty of Pharmacy, University of Khartoum, 11111 Qasr Street, P.O. Box 1996, Sudan; ^2^Graduate School of Biomedical Sciences, School of Pharmaceutical Sciences, Nagasaki University, 1-14 Bunkyo-machi, Nagasaki-shi 852-8521, Japan; ^3^Department of Pharmacognosy, Faculty of Pharmacy, Al-Azhar University, Assuit Branch, Assuit 71524, Egypt; ^4^The Medicinal and Aromatic Plants Research Institute (MAPRI), National Centre for Research, Mac Nimr Street, P.O. Box 2404, Khartoum, Sudan; ^5^Center for Industry, University and Government Cooperation, Nagasaki University, 1-14 Bunkyo-machi, Nagasaki-shi 852-8521, Japan; ^6^Kenya Research Station, Institute of Tropical Medicine, Nagasaki University, 1-12-4 Sakamoto, Nagasaki-shi 852-8523, Japan

## Abstract

A bioassay-guided fractionation of methanol extract of *Aristolochia bracteolata* whole plant was carried out in order to evaluate its antimicrobial activity and to identify the active compounds in this extract. Antibacterial and antifungal activities of methanol extract against gram-positive, gram-negative, and fungal strains were investigated by the agar disk diffusion method. Among the strains tested, *Moraxella catarrhalis* and sea urchin-derived *Bacillus* sp. showed the highest sensitivity towards the methanol extract and hence they are used as test organisms for the bioassay-guided fractionation. From this extract, aristolochic acid 1 (AA-1) has been isolated and has showed the greatest antibacterial activity against both standard strain and clinical isolates of *Moraxella catarrhalis* with equal minimum inhibitory concentration (MIC) and minimum bactericidal concentration (MBC) values of 25 and 50 *μ*g/mL. Modification of the AA-1 to AA-1 methyl ester completely abolished the antibacterial activity of the compound and the piperonylic acid moiety of AA-1 which suggested that the coexistence of phenanthrene ring and free carboxylic acid is essential for AA-1 antibacterial activity.

## 1. Introduction

*Moraxella catarrhalis* is a gram-negative, aerobic diplococcus human mucosal pathogen which causes middle ear infections in infants and children [1–3], and it is one of the three major causes of otitis media along with* Streptococcus pneumonia *and* Haemophilus influenzae *[4]. Although* Moraxella catarrhalis *is frequently found as a commensal of the upper respiratory tract, recently it has emerged as a genuine pathogen and is now considered an important cause of upper respiratory tract infections in healthy children and elderly people, lower respiratory tract infections in adults with chronic obstructive pulmonary disease [1, 5], and hospital-acquired pneumonia [6]. Amikacin, cefixime, fosfomycin, cefuroxime, cotrimoxazole, doxycycline, and erythromycin resistant strains of* Moraxella catarrhalis* were isolated and the widespread production of a *β*-1actamase enzyme renders the bacterium resistant to the penicillin [7–9].

This has led to the search for new and effective therapeutic alternatives among natural compounds. Plants remain an important source of diverse chemical entities which have been used as drugs or provide scaffolds from which new drugs have been derived [10]. The selection of a suitable candidate species for investigations can be done on the basis of long-term use by humans. This approach is based on an assumption that the active compounds isolated from such plants are likely to be safer than those derived from plant species with no history of human use [11].* Aristolochia* is an important genus in the family of* Aristolochiaceae* and is widespread across tropical Asia, Africa, and South America.* Aristolochia bracteolata* is commonly called “worm killer” in English due to supposed anthelmintic activity and trypanocidal effect [12]. It is used in traditional medicine as a gastric stimulant and in the treatment of cancer, lung inflammation, dysentery, and snakebites [13].* Aristolochiaceae* has been used by Sudanese people as analgesic, antiscorpion, and antisnake. It is also used in the treatment of tumors and malaria and for fevers [14], but its usage as an antimalarial is not recommended in its crude form.* Aristolochia bracteolata* showed a definite positive effect on wound healing, with significant increase in the level of powerful antioxidant enzymes. Its root and leaves were bitter and anthelmintic and are medicinally important. Almost every part of the plant has medicinal usage [15]. The whole plant was used as a purgative, antipyretic, and anti-inflammatory. It also possesses a potent antiallergic activity [16]. Organic solvent extracts of the plant showed antibacterial activities while the water extract showed antifungal activity [17]. The plant also showed promising antiarthritis activity [18]. Although* Aristolochia* has been used for thousands of years in many cultures for many indications due to its various pharmacological activities, it was later discovered that consuming these plants can certainly be dangerous. The genus of* Aristolochia* contains a naturally carcinogenic compound AA which has been shown to be the cause of so-called Chinese herb nephropathy or AA nephropathy [19, 20], and mutations in the cells of people who consume it, causes more mutations than two of the best-known environmental carcinogens: tobacco smoke and UV light [21, 22]. There are many cases of nephropathy reported in the literature caused by the systemic and long term application of Chinese snakeroot (*Aristolochia fangchi*); this highlighted the risk of using preparations which contain aristolochic acids [23].

Although* Aristolochia* is being known in many countries that is containing a toxic compound AA, but this has not stopped it from being a popular herbal remedy for thousands of years. It is still extensively used in India and in traditional Chinese medicine for slimming, menstrual symptoms, and rheumatism. It is also widespread used in Sudan and other African countries as one of the most effective herbal remedies for infectious diseases. Therefore, it was our objective to assess the potential antimicrobial activity of* Aristolochia bracteolata* using a bioassay-guided fractionation, in order to produce pure compound that can act as the lead compound in developing new, safe, and effective drug to replace the use of the harmful crude plant material.

## 2. Materials and Methods

### 2.1. Materials

#### 2.1.1. General

Sephadex LH-20 (Pharmacia Fine Chemical Co. Ltd) was used for column chromatography. Precoated silica gel plates (Merck, Kieselgel 60 F_254_, 0.25 mm) and precoated RP-18 F_254s_ plates (Merck) were used for thin-layer chromatography (TLC) analysis. High resolution FAB-MS and ESI-MS were recorded on JEOL JMS700N and JMS-100TD, respectively. ^1^H- and ^13^C-NMR, ^1^H-^1^H COSY, NOESY, HSQC, and HMBC spectra were recorded with a Unity plus 500 spectrometer (Varian Inc., U.S.A.) operating at 500 MHz for ^1^H and 125 MHz for ^13^C, respectively. ^1^H-NMR chemicals shifts are expressed in *δ* values referring to the solvent peak *δ*_H_ 2.49 for DMSO and coupling constants are expressed in Hz. ^13^C-NMR chemical shifts are expressed in *δ* values referring to the solvent peak *δ*_C_ 39.5 for DMSO. Piperonylic acid was purchased from commercial sources (TCI) and used without further purification.

#### 2.1.2. Plant Material

The plant material (whole) was collected in the period from (October to December 2012) from Khartoum state in Sudan. The plant was kindly identified and authenticated by the Taxonomist Dr. Haider Abdelgadir and Mr. Yahia Mohammed, Medicinal and Aromatic Plants Research Institute (MAPRI).

#### 2.1.3. Test Microorganisms

- Standard strains:* Moraxella catarrhalis* (GTC 01544),* Klebsiella pneumoniae* (ATCC 13883),* Escherichia coli* (K12),* Salmonella typhimurium* (ATCC 14028),* Streptococcus pyogenes* (ATCC 19615),* Streptococcus agalactiae* (ATCC 13813),* Staphylococcus epidermidis *(ATCC 12228),* Neisseria lactamicus* (ATCC 23970),* Enterobacter cloacae*, (ATCC 23355),* Bacillus subtilis* (ATCC 6633),* Staphylococcus aureus* (209P), and* Pseudomonas aeruginosa* (IFO 3445).
- Clinical strains*: Moraxella catarrhalis, Bacillus cereus, Aeromonas hydrophila, Salmonella typhi, Vibrio cholerae, *and* Yersinia enterocolitica.*

In addition to a sea urchin (*Anthocidaris crassispina*) derived* Bacillus* sp. which obtained from the Laboratory in Medical Plants Garden, Nagasaki University.

(c) Fungal strains: the fungal strains used were* Aspergillus niger* (NBRC 33023),* Penicillium crustosum* (NBRC 33015),* Schizophyllum commune* (NBRC 30749),* Trichophyton concentricum* (NBRC 31068), and* Candida albicans* (NBRC 10108).

### 2.2. Extraction of Plant Material

The air-dried powdered whole plant (200 g) was exhaustively extracted with cold maceration method with sufficient quantity of 70% methanol for 7 days at room temperature. The methanolic extract was passed through Whatman number 1 filter paper (Whatman England) and the concentrated extract (40 g) was digested with 100 mL distilled water and successively partitioned with* n*-hexane (4 × 400 mL), chloroform (3 × 400 mL), ethyl acetate (5 × 400 mL), and* n*-butanol (2 × 400 mL). Each fraction was concentrated under reduced pressure to a constant weight to give the corresponding* n*-hexane fraction (0.4 g), chloroform fraction (2 g), ethyl acetate fraction (0.7 g),* n*-butanol fraction (6 g), and aqueous fraction (30 g).

The most active fraction against* Bacillus* sp. and* M. catarrhalis* (chloroform fraction) was subjected to Sephadex LH20 column chromatography to give three subfractions (A-C). Fraction (B) was resubjected again to Sephadex LH20 to afford very active and pure compound AA-1 (150 mg).

### 2.3. Preparation of Methyl Ester of AA-1

To the solution of AA-1 (50 mg; 0.23 mmol) in dimethylformamide (DMF), 1 mL potassium carbonate was added (95 mg; 0.69 mmol). To the resulting suspension iodomethane was added (72 *μ*L; 1.15 mmol) and stirred for 8 hours. The reaction mixture was poured onto water (10 mL) and ethyl acetate (20 mL). The organic layer was washed with 1 M HCl (10 mL) three times and then with Brine (10 mL) once. The resulting solution was dried over magnesium sulphate and filtrated. After removal of solvent under reduced pressure, the residue was purified by silica-gel chromatography (chloroform-methanol) to afford the titled ester (88%). ^1^H-NMR (400 MHz, CDCl_3_, TMS, r.t.) *δ* (ppm): 3.88 (3H, s), 4.06 (3Hs), 6.38 (2H, s), 7.11 (1H, d,* J* = 7.8 Hz), 7.72 (1H, dd,* J* = 7.8 Hz, 8.0 Hz), 7.77 (1H, s), 8.70 (1H, d,* J* = 8.0 Hz), and 8.83 (1H, s).

### 2.4. Antimicrobial Activity

#### 2.4.1. Antibacterial Assay

The antibacterial activity was tested by agar disc diffusion assay [24]. Suspension of the tested bacteria (100 *μ*L of 10^8^ cfu/mL) was spread onto solid media plates. The sterile paper discs (6 mm in diameter) which were impregnated with the plant extract (1–4 mg) and pure compound (10–100 *μ*g) were placed aseptically over the bacterial culture on nutrient agar plates. After incubation at 37°C for 24 hours, the zone of inhibition around the discs was measured by millimeter scale. The experiment was replicated two times to confirm the reproducible results. Sterile, blank paper discs impregnated with only sterile solvents served as negative control each time.

#### 2.4.2. Antifungal Assay

The antifungal activity was determined by disk diffusion method [25, 26]. Fungi strains were inoculated on nutrient agar plates supplemented with 2% glucose. The sterile paper discs (6 mm in diameter) which were impregnated with individual extract were placed on the inoculated plates. These plates were incubated for 24–72 h at 25–28°C and the growth was evaluated visually by comparing a particular plate with the negative control plates (having only test fungi). The antifungal activity was evaluated by measuring the inhibition zone diameter (in millimeter) observed.

#### 2.4.3. Determination of MIC and MBC

The MIC and MBC values were determined by broth dilution method in accordance with CLSI methodology [27]. Bacterial strains were cultured for 24 h at 37°C on nutrient broth and then suspended in sterile distilled water to give a final inoculum concentration of 1.5 ± 1.0 × 10^3^ cfu/mL. Dilutions ranging from 1.56 to 400 *μ*g/mL of the compound were prepared in tubes including broth and DMSO 10% (v/v), in addition to one negative control (broth + DMSO 10% v/v + test microorganism) to ensure that the final concentration of DMSO in the assays did not interfere with the bacterial growth and one sterility control (broth + DMSO 10% v/v + test compound). A 100 *μ*L suspension of test microorganism was added to individual tubes and incubated at 37°C for 24 h. The MIC of the compound was defined as the lowest concentration that inhibited the visible bacterial growth and the MBC was defined as the lowest concentration that prevented the growth of the organism after subculture onto antibiotic-free plates. Afterward results were confirmed by two trained laboratory personnel.

## 3. Results and Discussion

### 3.1. Antimicrobial Activity

Initial steps in new drug discovery involve identification of new chemical entities, which can be either sourced through chemical synthesis or can be isolated from natural products through biological activity guided fractionation. Bioassay-guided fractionation of the identified plant may lead to standardized extract or isolated bioactive lead compounds as the new drug [11].

The whole plant of* Aristolochia bracteolata* was extracted successively with MeOH and subjected to liquid-liquid fractionation with* n*-hexane, chloroform, ethyl acetate, and* n*-butanol. The resulting fractions were tested for antibacterial and antifungal activities. The crude extract and chloroform fraction were significantly active against sea urchin-derived* Bacillus *sp. and both standard strain and clinical isolates of* Moraxella catarrhalis* and were moderately active against* S. aureus, B. subtilis,* and* Ps. aeruginosa*. The* n*-hexane fraction had moderate activity against* S. aureus* and* B. subtilis* while ethyl acetate fraction showed moderate activity against* Ps. aeruginosa* and* B. subtilis*. All fractions were active against sea urchin-derived* Bacillus sp.* (Table 1). The crude extract failed to inhibit the growth of all test fungi in addition to the following bacterial strains:* Klebsiella pneumoniae*,* Escherichia coli*,* Salmonella typhimurium*,* Streptococcus pyogenes*,* Streptococcus agalactiae*,* Staphylococcus epidermidis*,* Neisseria lactamicus*,* Enterobacter cloacae*,* Bacillus cereus, Aeromonas hydrophila, Salmonella typhi, Vibrio cholerae, and Yersinia enterocolitica.*

**Figure 1:**
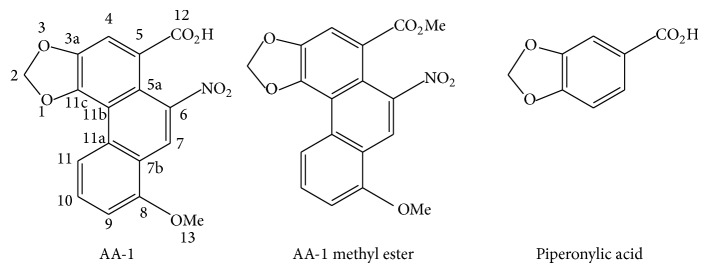
Structure of AA-1, AA-1 methyl ester, and piperonylic acid.

The chloroform soluble fraction was therefore selected for further chromatographic separations and resulted in the isolation of known compound AA-1 (Figure 1). AA-1 showed strong activity against* Moraxella catarrhalis* (standard strain and clinical isolates) and sea urchin-derived* Bacillus sp.* (Table 2), with equal MIC and MBC values of 25 and 50 *μ*g/mL. Both the piperonylic acid moiety of AA-1 (Figure 1) and AA-1-methyl ester showed no activity against bacteria (Table 2), which suggests that the coexistence of phenanthrene ring and free carboxylic acid is essential for AA-1 antibacterial activity.

### 3.2. Structure Elucidation of AA-1

Bioguided fractionation of methanolic extract of* Aristolochia bracteolata* led to isolation of AA-1 and its structure was elucidated by interpretation of its NMR and MS data and by comparison with those reported in the literature. Electrospray ionization mass spectrometry (ESIMS) showed pseudomolecular ion signal at* m/z* 364.03 [M + Na]^+^ and high resolution fast atom bombardment mass spectrometry (HR-FABMS) afforded M+1 ion signal at* m/z* 342.0620 which was corresponding to the molecular formula C_17_H_12_NO_7_ (calculated for 342.06138). ^1^H NMR and ^3^C-NMR spectra were matched with those of AA-1 which were previously reported [28]. The correlation among each NMR signal was confirmed by 2D HSQC and HMBC. Two weak signals which did not show any correlation with proton in HSQC and HMBC spectroscopy were considered as quaternary carbons at positions 5 (*δ* 124.3 ppm) and 6 (*δ* 143.5 ppm), respectively.

Laboratories of the world have found literally thousands of phytochemicals which have inhibitory effects on all types of microorganisms* in vitro*. More of these compounds should be subjected to animal and human studies to determine their effectiveness in whole-organism systems, including in particular toxicity studies and an examination of their effects on beneficial normal microbiota [10].

In spite of the fact that herbal remedy is a mixture of many chemicals in unknown doses and might result in unpleasant side effects, many people believe that treatments that are natural are somehow magically safe and effective.

*Aristolochia* is used in traditional medicine for the treatment of various diseases [13, 15], including those associated with bacteria. This study showed clearly that the excellent effect of* Aristolochia* in treating such diseases is due to the toxic compound AA-1. Although AA-1 is highly effective in killing* M. catarrhalis*, it is ineffective against the other microorganisms tested. This highlights the importance of* M. catarrhalis* in discovering the cellular target of AA-1 and the mechanism of AA-1 toxicity. The widespread use of* Aristolochia* is not sufficient to ensure that it is effective or even that it is safe. Therefore, hit-to-lead exploration is necessary to identify related compounds with low toxicity, low cost, and improved potency that can replace the use of the harmful crude plant material.

It is impossible to ban the use of these remedies, especially in the rural areas in Sudan and other African countries; therefore, we strongly recommend educating the public of the risks versus the benefits of* Aristolochia* and gradually replacing them with either economical new drugs or standardized extracts and homogenous batches of other plant material with known levels of safe active constituents.

## 4. Conclusion

Using bioassay-guided fractionation technique, the present study directly linked the antibacterial activity of* Aristolochia bracteolata* to the AA-1. Although AA-1 had strong activity against* M. catarrhalis*, it had a narrow spectrum of activity than expected based on the activity of the crude extract from which it was isolated or from its traditional usage. This may be the result of synergism between different compounds in the complex extracts or may be due to placebo effect.

## Figures and Tables

**Table 1:** Antibacterial activity of crude plant extract and fractions.

Diameter of zone of inhibition (mm)					
Microorganism	Crude extract	*n*-Hexane	Chloroform	Ethyl acetate	*n*-Butanol
*B. subtilis *	15	12	16	12	—
Marine *Bacillus sp. *	25	18	25	20	15
*S. aureus *	15	12	11	—	—
*Ps. aeruginosa *	10	—	11	11	—
*M. catarrhalis *	12	—	14	9	—

**Table 2:** Antibacterial activity of AA-1 and its derivatives.

Diameter of zone of inhibition (mm)				
Microorganism	AA-1	AA-1 methyl ester	Piperonylic acid	Ciprofloxacin
Marine *Bacillus sp. *	12	—	—	20
*M. catarrhalis *T	12	—	—	20
*M. catarrhalis *1 CI	11	—	—	18
*M. catarrhalis* 2 CI	12	—	—	19
*M. catarrhalis* 3 CI	12	—	—	20
*M. catarrhalis* 4 CI	11	—	—	20
*M. catarrhalis *5 CI	12	—	—	19

T: type strain; CI: clinical isolate.
